# HEARING THE VOICE OF OLDER MIGRANTS WITH DEMENTIA: A DECOLONIAL APPROACH OF PARTICIPATIVE ACTION RESEARCH

**DOI:** 10.1093/geroni/igac059.978

**Published:** 2022-12-20

**Authors:** Saloua Berdai Chaouni

**Affiliations:** University College Karel de Grote Antwerp, Antwerp, Antwerpen, Belgium

## Abstract

Gerontological research has been proven not always to succeed in engaging older migrants and their families. Various attempts are made to give voice to this under-researched population. Qualitative methods like participative action research (PAR) have been put forward as a way to engage this population. However, this approach does not always succeed to achieve this goal. Drawing on insights from decolonial frameworks, we present a learning process in engaging older migrants with and without dementia and their family members in developing a migration-sensitive reminiscence approach as a psycho-social intervention for older migrants with dementia. The emphasis of decolonial perspectives on seeing this population as the “Knower”, deep reflection on own coloniality of mind as a researcher while critically looking at exclusive aspects of epistemology offers a supporting gaze to reshape PAR as an approach where this population is not only given voice but also heard.

